# Ethnic Differences in Physiological Responses to Fear Conditioned Stimuli

**DOI:** 10.1371/journal.pone.0114977

**Published:** 2014-12-12

**Authors:** Karen G. Martínez, José A. Franco-Chaves, Mohammed R. Milad, Gregory J. Quirk

**Affiliations:** 1 Department of Psychiatry, University of Puerto Rico School of Medicine, San Juan, Puerto Rico; 2 Department of Anatomy & Neurobiology, University of Puerto Rico School of Medicine, San Juan, Puerto Rico; 3 Department of Psychiatry, Massachusetts General Hospital and Harvard Medical School, Charlestown, MA, United States of America; University of Tasmania, Australia

## Abstract

The idea that emotional expression varies with ethnicity is based largely on questionnaires and behavioral observations rather than physiological measures. We therefore compared the skin conductance responses (SCR) of Hispanic (Puerto Rican) and White non-Hispanic subjects in a fear conditioning and fear extinction task. Subjects were recruited from two sites: San Juan, Puerto Rico (PR), and Boston, Massachusetts (MA), using identical methods. A total of 78 healthy subjects (39 from PR, 39 from MA) were divided by sex and matched for age and educational level. Females from the two sites did not differ in their SCRs during any experimental phase of fear conditioning (habituation, conditioning, or extinction). In contrast, PR males responded significantly to the conditioned stimulus than MA males or PR females. Subtracting ethnic differences observed during the habituation phase (prior to conditioning) eliminated differences from subsequent phases, suggesting that PR males are elevated in their response to novelty rather than fear learning. Our findings suggest that, in addition to sex differences, there are ethnic differences in physiological responses to novel stimuli at least in males, which could be relevant for the assessment and treatment of anxiety disorders.

## Introduction

It is widely accepted that the emotions of fear and anxiety are modulated by ethnicity and cultural background [1], [2], [3]. Furthermore, the prevalence of anxiety disorders appears to differ between ethnic groups [4], [5]. Reports of ethnic differences, however, could be compromised by the limited validity of cross-ethnic psychological measures used to assess anxiety [5]. Self-reports of symptoms are difficult to interpret because they can vary with education level or other socioeconomic factors [6]. For example in Hispanics, physical anxiety symptoms more accurately predict Generalized Anxiety Disorder (GAD) than thought-based questionnaires assessing worrying [7], [8].

Assessing physiological markers could be a way to overcome limitations of self-reports in the study of ethnic differences in anxiety. Several physiological measures, such as heart rate, respiratory rate, blood pressure, and skin conductance response (SCR) correlate with verbal reports of fear [9]. The SCR has been frequently assessed in experimental fear conditioning, in which a visual stimulus is paired with a mild shock [10], [11]. SCRs recorded during conditioning and extinction can vary between different anxiety disorders. For example, overgeneralization of conditioned stimuli is seen in social phobia, [12] whereas deficient recall of extinction is seen in post-traumatic stress disorder (PTSD) [13].

To determine the extent to which Hispanic ethnicity modulates conditioned SCRs, we compared two groups of healthy subjects: Puerto Rican subjects from San Juan, Puerto Rico (PR) (n = 39), and White Non-Hispanic subjects from Boston, MA (MA) (n = 39), during experimental fear conditioning and extinction. Because previous studies have shown sex differences in extinction [14], we also compared male and female subjects in the PR and MA groups.

## Methods

### Participants

Healthy subjects in Puerto Rico (PR) and in Massachusetts (MA) were recruited from the local community via advertisements. Exclusion criteria included a history of neurological conditions, current psychoactive medications, or Axis I diagnosis within the past 6 months. A Structured Clinical Interview for DMS-IV (SCID-I-RV) was used to confirm the absence of a psychiatric diagnosis. The final sample consisted of 78 subjects matched for age and sex between the two sites (20 PR females, 20 MA females, 19 PR males and 19 MA males) ranging in age from 18–34 (Mean age females  = 24.2±2.95 years; Mean age males  = 25±4.01 years). Written informed consent was obtained from all participants in accordance with the requirements of the Institutional Review Board at the University of Puerto Rico, School of Medicine and at Harvard Medical School.

### Demographics and Psychological Tests

Data was gathered on educational level, anxiety symptoms and personality characteristics across sites. Questionnaires for the PR site were previously validated for use with Spanish speaking subjects.

- **Anxiety symptoms scales.** We used the State Trait Anxiety Inventory (STAI) to measure anxiety symptoms. The STAI is a self-report instrument that differentiates between the temporary condition of state anxiety and the longstanding quality of trait anxiety [15]. It has 20 items for assessing trait anxiety and 20 for state anxiety. Internal consistency coefficients for the scale have ranged from α = .86 to.95 [15]. The Spanish version of the STAI in Puerto Rico yielded a high internal consistency in both State α = .83 to.92 and Trait α = .86 to.92 [16].
- **NEO Five Factor Inventory (NEO-FFI).** The NEO-FFI is a 60-item self-report measure of personality traits across five dimensions: neuroticism, extraversion, agreeableness, conscientiousness and openness to experience [17]. Psychometric studies of this version of the Neo have not been reported with a Puerto Rican sample, however the long version of Neo (NEO-PI-R) has reported moderate to high consistency with Puerto Rican samples [18]. Raw scores for each personality dimension were calculated and subsequently transformed into T-scores (M = 50, SD = 10), to adjust for sex differences.

### Fear Conditioning and Extinction

**Figure 1:**
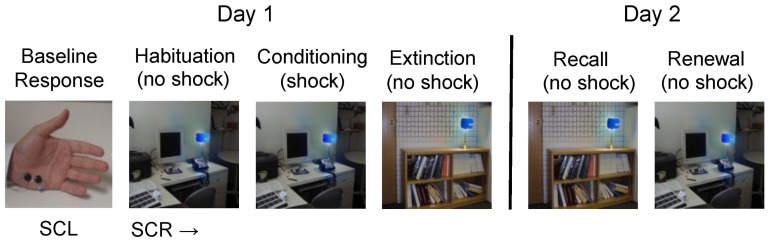
Schematic representation of research protocol. Electrodes measuring skin conductance response (SCR) are placed on the palm of the non-dominant hand while shock electrodes were placed on the dominant hand fingers. Subjects first encounter all the images without any shock (habituation) and then in one of the contexts (office) the blue light will be paired with a shock while the red light receives no shock (conditioning). The other context (library) is then shown with no shocks neither in the blue or red light (extinction). Approximately 24 hours later, subjects return and their SCR is measured while seeing the extinction context with no shock (recall) and the conditioning context with no shock (renewal). SCL =  skin conductance level, SCR  =  skin conductance response.

We used the same fear conditioning protocol at the two sites, as previously described by Milad and coworkers [10], in which subjects were tested over two consecutive days (see Fig. 1). On day 1, subjects received habituation, conditioning, and extinction trials. Habituation consisted of 8 trials in which the conditioned stimuli (red or blue desk lights) were presented against two separate contextual backgrounds (library or office), without any shock. The context stimulus appeared 6 sec prior to the onset of the desk light, with the light with the context then lasting for 12 sec, after which the screen went black. The average inter-trial interval was 16 sec (range 12–21 sec). The habituation phase was immediately followed by the conditioning phase, in which one of the two desk lights (e.g., red) served as the CS+ and was paired with a mild shock that started immediately after CS+ offset and lasted 0.5 sec. Electrodes were placed on the second and third fingers of the dominant hand. Conditioning occurred within a specific context (e.g., office) (see Fig. 1). The alternative desk light (e.g., blue, CS-) was presented without any shock. The color of the CS+ (blue or red), as well as the context (library or office), were counterbalanced across subjects. The electric current was generated by a Coulbourn transcutaneous aversive finger stimulator (E13-22) powered by a 9V dry cell battery. The intensity of the current was chosen by each participant to be “highly annoying, but not painful”. The electrodes remained attached to the subjects' fingers during all phases of the experiment, and subjects were instructed prior to each phase that they “may or may not receive a shock”. Subjects were given 10 trials of conditioning (5 CS+ and 5 CS-), followed a few minutes later by 20 trials of extinction (10 CS+ and 10 CS-), in which the CS+ and CS- were presented without shocks in the alternate context.

On Day 2, subjects were brought back to the lab to assess their retention of extinction. Subjects were shown the context stimulus alone, followed 6 sec later by the CS+ without any shock. Extinction memory was tested in two phases: recall and renewal, each consisting of 10 trials (5 CS+ and 5 CS-). During the recall phase, the CS+ was presented in the extinction context (recall of safety), whereas during the renewal phase, the CS+ was presented in the conditioning context (recall of danger). The order of testing (recall vs. renewal) was counterbalanced across subjects.

### Physiological Measures

The baseline skin conductance level (SCL) consisted of the average skin conductance during the 5 seconds prior to the first habituation trial. Subjects' unconditioned response (UCR) to the shock consisted of the change in SCL within 3 sec after shock, prior to conditioning. Subjects' skin conductance response (SCR) was their response to the 12 sec light stimulus (CS+, CS), minus their response to the 6 sec context stimulus preceding the light, as previously described [10]. We applied a criterion for conditioning requiring that subjects' SCR to the CS+ exceeded 0.05 µS, in 2 or more conditioning trials [10].

### Statistical analyses

Subjects' demographics and psychological test scores were compared between the two sites using a ttest. Group differences and sex differences in SCRs between males and females at the two sites were compared with ANOVAs with repeated measures. A three-factor ANOVA (group vs. sex vs. trial) were performed for each phase of the experiment, to test for main effects and interactions. Post-hoc tests (Tukey) were used to follow up on significant interactions (Statistica, version 19.0).

## Results

### Psychological tests

Healthy subjects at both sites scored within the normal range on all psychological tests (Table 1). There were no ethnic differences in mean age or educational level (see Table 1). Females from PR and MA did not differ in state anxiety, however, PR males showed somewhat lower state anxiety compared to MA males (PR males  = 26.17, MA males  = 30.13; t_(1,32)_ = 2.113; p = 0.042). In the NEO-FFI personality scale, males at the two sites showed no differences, however PR females showed significantly higher levels of neuroticism (MA females  = 37.47, PR females  = 49.25; t_(1,34)_ = −4.288; p = 0.001) and lower levels of agreeableness (MA females mean = 59.17, PR females mean = 48.10; t_(1,34)_ = 7.536; p = 0.008) than MA females (Table 1). There were no ethnic differences in trait anxiety.

**Table 1:** Demographics and Psychological Tests Scores of sample divided by sex and site with ttest comparison between PR and MA (ethnic) and between females and males (sex) at each site.

Demographic Data	PR females Mean (s.d.)	MA females Mean (s.d.)	PR males Mean (s.d.)	MA males Mean (s.d.)	ttest Ethnic Comparison (PR vs MA)		ttestSex Comparison (females vs males)	
					females	males	PR	MA
Age	25 (3)	24 (3)	26 (3)	25 (4.01)	0.461	0.515	0.358	0.139
Educational level	16.60 (2)	16.34 (1.5)	17 (3)	16.95 (2.01)	0.661	0.657	0.827	0.303
**NEO-FFI**								
Neuroticism	**49.25 (8.42)**	**37.47 (7.37)**	43.00 (2.70)	**45.13 (2.45)**	**<0.001**	0.589	0.063	**0.023**
Extraversion	57.95 (8.11)	60.88 (8.80)	55.53 (2.15)	57.27 (1.89)	0.299	0.575	0.393	0.364
Openness	54.3 (10.80)	60.71 (9.95)	56.68 (2.65)	63.64 (1.56)	0.071	0.053	0.509	0.356
Agreeableness	**48.1 (9.81)**	**59.17 (14.2)**	45.58 (2.78)	**49.6 (3.11)**	**0.008**	0.368	0.478	**0.014**
Conscientiousness	48.75 (12.0)	53.65 (12.4)	47.84 (1.81)	49.93 (1.91)	0.231	0.469	0.783	0.195
**STAI**								
Trait Anxiety	32.6 (6.85)	31 (6.89)	29.63 (1.95)	33.24 (1.28)	0.509	0.148	0.237	0.201
State Anxiety	29.9 (6.91)	30.3 (6.89)	**26.17 (0.92)**	**30.13 (1.39)**	0.897	**0.042**	0.068	0.721

### Analysis of shock level, skin conductance level, and unconditioned responses

There was no difference in the level of shock chosen between the PR and MA males (t_(1,36)_ = 1.224, p = 0.229). In contrast, MA females chose significantly lower levels of shock than PR females (MA females mean = 1.68 mv; PR females mean = 2.87 mv; t_(1,38)_ = 3.81, p = 0.004). Comparing across sex, the PR males and females did not show any significant difference in shock level (t_(1,37)_ = -0.613, p = 0.543), but MA females chose significantly lower shock levels than MA males (MA males mean  = 3.07 mv; MA females mean  = 1.68 mv; t_(1,37)_ = 4.521, p<0.001) (Table 2).

**Table 2:** Shock level chosen and Unconditioned response of sample divided by sex and site with ttest comparison between PR and MA (ethnic) and between females and males (sex) at each site.

Shock Measures	PR females Mean (s.d.)	MA females Mean (s.d.)	PR males Mean (s.d.)	MA males Mean (s.d.)	ttest Ethnic Comparison (PR vs MA)		ttest Sex Comparison (females vs males)	
					females	males	PR	MA
Shock level chosen	**2.87 (1.11)**	**1.68 (0.87)**	2.66(0.99)	**3.07(1.05)**	**0.004**	0.229	0.543	**<0.001**
Unconditioned response (UCR)	0.85 (0.70)	0.86 (0.99)	1.01 (0.58)	1.36 (1.12)	0.992	0.230	0.481	0.147

**Figure 2:**
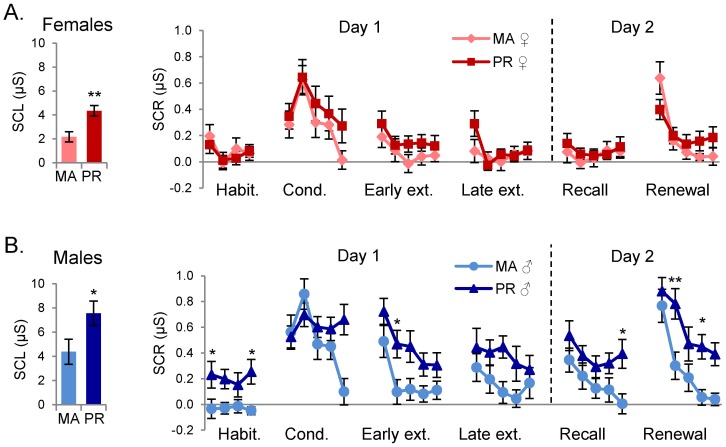
Baseline skin conductance level (SCL) and skin conductance responses (SCR) to CS+ across all experimental phases in males and females at both sites. A. SCL and responses to CS+ in females in Massachusetts (MA) and Puerto Rico (PR). B. SCL and responses to CS+ in males in Massachusetts (MA) and Puerto Rico (PR). Habit. = Habituation, Cond. = Conditioning, Ext. = Extinction, μS  =  microsiemens. *p<0.05; **p<0.01.

The baseline (pre-conditioning) skin conductance level (SCL) was significantly higher in the PR sample compared to the MA sample. This was true for females (MA females  = 2.17; PR females  = 4.34; t_(1,38)_ = 3.56, p = 0.001) and males (MA males  = 4.38; PR males  = 7.55; t_(1,36)_ = 2.19, p = 0.034) (see Fig. 2), and may be due to the high atmospheric humidity in PR. In both PR and MA subjects, SCL values were significantly larger in males than in females (PR males  = 7.55; PR females  = 4.34; t_(1,37)_ = −2.972; p = 0.005; MA males  = 4.38; BA females  = 2.17; t_(1,37)_ = 4.381; p = 0.019). There were no significant differences in the unconditioned response (UCR, the response to the shock stimulus alone) across ethnicity or sex (Table 2).

### Analysis of habituation, conditioning, and extinction phases on Day 1

**Figure 3:**
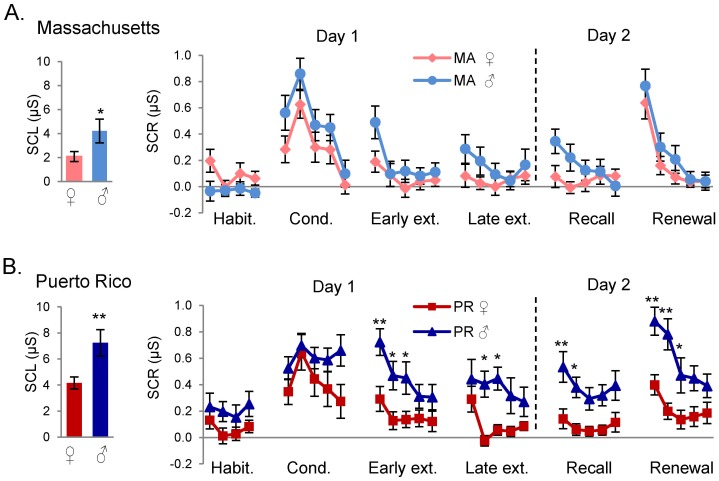
Sex differences at both sites in skin conductance responses (SCR) to CS+ across all experimental phases. A. Responses to CS+ in males and females in Massachusetts (MA). B. Responses to CS+ in males and females in Puerto Rico (PR). Habit. = Habituation, Cond. = Conditioning, Ext. = Extinction, μS  =  microsiemens. *p<0.05; **p<0.01.

We first analyzed the responses to the conditioned stimulus paired with shock (CS+). During the habituation phase, ANOVA showed a main effect of ethnicity (F_(1,74)_ = 5.247, p = 0.025) but not sex (F_(1,74)_ = 0.065, p = 0.7997) and there was a significant interaction between ethnicity and sex (F_(1,74)_ = 7.958, p = 0.006),. Post-hoc tests showed that PR males were significantly higher than MA males in trial 4 of habituation (p = 0.033) (Fig. 2A). During conditioning, there was no main effect of ethnicity (F_(1,74)_ = 2.071, p = 0.154), but there was a significant effect of sex (F_(1,74)_ = 5.289, p = 0.024) with no significant interaction between sex and ethnicity (F_(1,74)_ = 0.005, p = 0.941). Post-hoc tests did not reveal any significant trial differences in conditioning between sexes (Fig. 3).

During early extinction, there were significant main effects of sex (F_(1,74)_ = 12.568, p = 0.001) and ethnicity (F_(1,74)_ = 10.745, p = 0.002), but no interaction. Post-hoc analysis showed that males were significantly higher than females in trial 1 (p<0.001) (Fig. 3), and that PR subjects were significantly higher than MA subjects in trial 3 (Fig. 2A). During the late extinction phase, there continued to be main effects of sex (F_(1,74)_ = 14.762, p<0.001) and ethnicity (F_(1,74)_ = 6.475, p = 0.013), but no interactions. Post-hoc analysis showed that males were significantly higher than females in trial 2 (p = 0.007) (Fig. 3).

We next analyzed responses to the conditioned stimulus that was never paired with shock (CS-). There was no effect of sex, ethnicity or interaction in CS- responses during habituation or early extinction. During conditioning, there was a main effect of ethnicity (F_(1,74)_ = 5.128, p = 0.003) with no significant differences during post-hoc tests and no main effect of sex or interaction. In the late extinction phase, there was no main effect of sex or ethnicity, but there was an interaction effect (F_(1,74)_ = 4.513, p = 0.037) with no significant post-hoc tests. Differential learning (the difference between CS+ and CS-) did not differ between PR and MA subjects in any phase, in either males or females.

In summary, during Day 1, PR and MA females showed comparable SCRs (Fig. 2B) while PR males showed larger habituation responses than MA males (Fig. 2). An ethnic difference was also found in early extinction with the PR sample showing higher SCR in trial 3 than the MA sample (Fig. 2). There was also a sex difference in responses during extinction with males showing higher SCR than females during trial 1 of early extinction and trial 2 of late extinction (Fig. 3).

### Analysis of extinction-recall and renewal phases on Day 2

The day following conditioning and extinction, subjects returned to the laboratory to test for recall of extinction memory (CS+ presented in the extinction context), as well as renewal of fear (CS+ presented in the conditioning context). Recall and renewal tests were administered across subjects in a counterbalanced order. During recall, ANOVA revealed significant main effects of sex (F_(1,74)_ = 18.936, p<0.001), ethnicity (F_(1,74)_ = 7.275, p = 0.009), and interaction effects (F_(1,74)_ = 4.183, p = 0.044). PR females and MA females continued to exhibit equivalent responses throughout Day 2 (see Fig. 2). Post-hoc analysis showed that males were significantly higher than females in trials 1 (p<0.001) and 2 (p = 0.003) (Fig. 3). There were no significant ethnic differences within trials (Fig. 2). The sex difference was only seen in the PR sample, with PR males continuing to be significantly higher PR females in trial 1 (p = 0.009) (Fig. 3B).

During the renewal phase, ANOVA revealed a significant main effect of sex (F_(1,74)_ = 16.0067, p<0.001) and ethnicity (F_(1,74)_ = 8.949, p = 0.003), as well as an interaction effect (F_(1,74)_ = 6.474, p = 0.013). Post-hoc analysis showed that males were significantly higher than females in trials 1 (p = 0.002) and 2 (p<0.001), and that PR subjects were significantly higher than MA subjects in trials 2 (p = 0.024) and 4 (p = 0.024). The post-hoc analysis revealed that there was a significant difference between PR and MA males, with PR male responses higher in trials 2 (p = 0.001) and 4 (p = 0.033) (Fig. 2A). Furthermore, PR males were significantly higher than PR females in trials 1 (p<0.001) and 2 (p<0.001) (Fig. 3A).

In terms of responses to CS-, during recall, there was a main effect of sex (F_(1,74)_ = 9.106, p = 0.003) and an interaction effect (F_(1,74)_ = 7.921, p = 0.006), but no main effect of ethnicity (F_(1,74)_ = 1.341, p = 0.251). Post-hoc tests showed no individual trial differences. During renewal, there were no main effects or interactions in the CS- responses. For differential learning, there was a sex effect (F_(1, 74)_ = 4.547, p = 0.036) during recall, with no ethnic (F_(1, 74)_ = 3.250, p = 0.075) or interaction (F_(1, 74)_ = 0.003, p = 0.953) effects. Post-hoc tests showed no significant individual differences between sexes. For differential responses in renewal there was a sex (F_(1,74)_ = 9.724, p = 0.0026) and ethnic (F_(1, 74)_ = 4.268, p = 0.042) main effect as well as an interaction effect (F_(1, 74)_ = 5.727, p = 0.019). Post-hoc tests did not show significant differences for individual trials between sexes or ethnicity.

**Figure 4:**
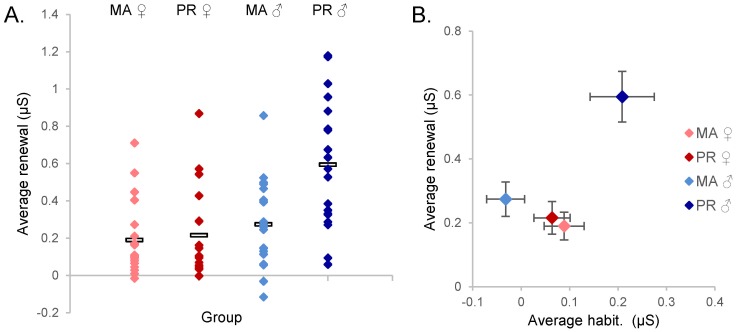
Summary of SCR differences in PR males. A. Increased SCR during renewal is seen in PR males when compared to PR females, MA males and females. B. PR males show both increased habituation and renewal when compared to PR females, MA males and females.

Summarizing, Day 2 showed only a sex difference in the PR sample during recall of extinction. During renewal of conditioning, there were both sex and ethnic differences, with PR males showing significantly higher SCRs during this phase than the three other groups (Fig. 4A).

### Subtraction of habituation phase responses

Because PR males showed larger SCR responses during the habituation phase (prior to conditioning), it is possible that differences observed in the conditioning and extinction phases simply reflected this elevated initial response. To address this, we normalized conditioning and extinction phases to the habituation phase by subtracting the average habituation SCR (trials 1–5) from each subsequent trial, for each subject. Subtracting habituation phase responses caused SCRs in PR males to be equivalent to MA males in all subsequent phases (S1A Figure). Indeed, this manipulation eliminated all significant main effects of ethnicity, suggesting that elevated CS+ responses in PR males may be due to an elevated response in habituation, rather than heightened fear learning. This is supported by the absence of any group differences in differential responding (see above).

In contrast to effects of ethnicity, main effects of sex persisted despite normalizing conditioning and extinction phases to the habituation phase (S1B Figure). In conditioning, there was a main effect of sex (F_(1,74)_ = 4.601, p = 0.035) with no significant post-hoc tests. In early extinction, the main effect of sex persisted (F_(1,74)_ = 10.582, p = 0.002) with post-hoc tests confirming that males showed higher SCRs than women during trial 1 (p<0.001) and trial 3 (p<0.049). There was also a main effect of sex in late extinction (F_(1,37)_ = 7.856, p = 0.006) with males showing higher SCRs during trial 2 (p = 0.013). The sex difference was also seen on day 2 with a main effect during recall (F_(1,74)_ = 10.461, p = 0.002) and post-hoc tests showing higher SCRs in males during trial 1 (p<0.001) and trial 2 (p = 0.004). The sex effect persisted in renewal (F_(1,37)_ = 10.319, p = 0.002) and males showed higher SCR during trials 1 (p = 0.002) and 2 (p<0.001). Thus, unlike ethnic differences, sex differences could not be attributed to elevated responses in the habituation phase.

In conclusion, PR males showed larger SCRs than MA males or females at either site, during habituation and renewal phases (Fig. 4B). Increased responses in habituation could account for the ethnic differences between the PR and MA males in subsequent phases, but could not account for the sex differences in renewal.

## Discussion

Our results indicate a pronounced ethnic difference in physiological fear responses, comparing subjects in PR vs. MA. This difference was observed only in males, with PR males showing higher SCRs than MA males or females at both sites. We are able to rule out several factors that could potentially account for these differences: 1) The experimental procedures at the two sites did not significantly differ, given that females showed no ethnic differences in SCR. 2) Increased skin conductance level (SCL), potentially due to climate differences, was observed in both males and females, even though females showed no differences in SCRs. 3) The shock levels selected by subjects were equivalent in PR males and MA males, as was the unconditioned responses to shock (UCR). 4) PR males did not show increased anxiety, as levels of state anxiety were lower in PR males compared to MA males, and no ethnic differences in males were found in trait anxiety or neuroticism. Thus, the SCR difference we observed in male subjects at the two sites appears to be due to ethnicity.

Because PR males showed larger responses during the habituation phase (prior to conditioning), and because subtracting this difference normalized subsequent phases, the excessive SCR in PR males does not reflect increased conditioning or deficient extinction. Indeed, there were no ethnic differences in the differential response, which is the best indicator of associative learning. Instead, increased responding during the habituation phase in PR males could be due to an increased response to novelty, which persisted in later phases. A failure to habituate to novel stimuli is correlated with inhibited temperament, a known risk factor for anxiety disorders [19]. Deficient habituation to repeated stimuli has also been identified as a physiological marker of post-traumatic stress disorder (PTSD) [20]. Thus, this physiological response in PR males may indicate a risk for the development of anxiety disorders.

PR males also showed larger SCRs than PR females. No such sex difference was observed in the MA group, in contrast to prior studies showing that males have higher conditioned SCRs than females [21], [22]. Sex differences in conditioned SCRs have been inconsistent, [23] perhaps due to variability in women's menstrual cycle, which was not evaluated in our study. The larger SCR in renewal in PR males persisted after subtraction of habituation SCRs (Fig. 4), suggesting that excessive renewal was not simply an effect of novelty. This may have important clinical implications, as renewal of extinguished fear is thought to model relapse after treatment with exposure therapy [24].

There are several limitations in our study. We did not address specific factors that could potentially modulate SCRs such as genes, environment, or some interaction between the two. To fully evaluate the effect of environment, an additional experimental group would be needed, such as MA males living in PR or PR males living in MA. We did not fully assess socioeconomic status: only data on educational level was obtained. Another potential factor not assessed in our study is the presence of childhood trauma, which has been associated with heightened skin conductance [25]. Finally, cultural factors determining how males and females are expected to behave [26] could also modulate SCRs. Because we used a relatively small sample of subjects recruited within a university setting, our findings may not be generalizable to all males in Puerto Rico. It would be important to replicate these results with a larger, more varied sample.

Our subject pool consisted of healthy subjects, however, our findings could be related to risks for anxiety disorders. A previous study has shown that male veterans from PR have a higher risk for the development of PTSD, compared to white non-Hispanic, African American, and other Hispanic veterans [27]. It has been suggested that the higher rates of PTSD in PR veterans may be a consequence of over-reporting of arousal symptoms in Hispanics [6], however, our findings suggest a possible physiological basis. The inclusion of physiological measurements such as SCRs may be important for assessing the effect of ethnicity on anxiety. Our findings may also be relevant for the treatment of PTSD, as elevated fear responses to traumatic stimuli could interfere with the progress of extinction-based therapies.

## Supporting Information

S1 Figure **Differences in skin conductance responses (SCR) when habituation is subtracted from all subsequent phases.** A. Average habit. subtracted from responses to CS+ in males in Massachusetts (MA) and Puerto Rico (PR). B. Average habit. subtracted from responses to CS+ in females and males combined from both sites (MA andPR). Habit. = Habituation, Cond. = Conditioning, Ext. = Extinction, μS  =  microsiemens. *p<0.05; **p<0.01.
(TIFF)

S1 File (XLSX)
