# Supplementary figures and images for: Ethnic Differences in Physiological Responses to Fear Conditioned Stimuli
Source: PLoS One. 2014 Dec 12;9(12):e114977. doi: 10.1371/journal.pone.0114977 (PMC4264849; doi:10.1371/journal.pone.0114977)

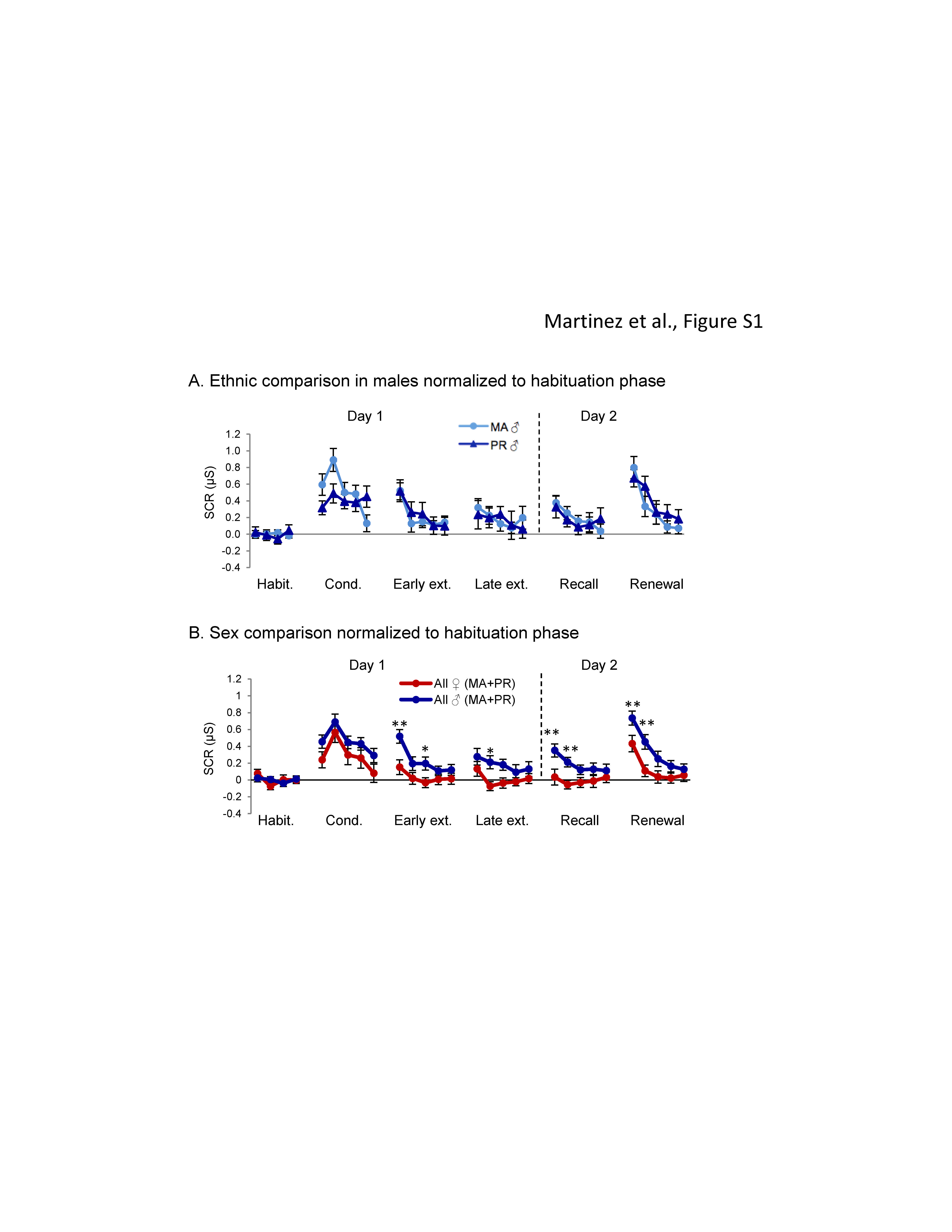

Supplement: S1 Figure — Differences in skin conductance responses (SCR) when habituation is subtracted from all subsequent phases. A. Average habit. subtracted from responses to CS+ in males in Massachusetts (MA) and Puerto Rico (PR). B. Average habit. subtracted from responses to CS+ in females and males combined from both sites (MA andPR). Habit. = Habituation, Cond. = Conditioning, Ext. = Extinction, μS = microsiemens. *p<0.05; **p<0.01. (TIFF) [file pone.0114977.s001.tiff]
